# Uptake of new antidiabetic medications in three emerging markets: a comparison between Brazil, China and Thailand

**DOI:** 10.1186/s40545-014-0020-4

**Published:** 2015-02-16

**Authors:** Christine Y Lu, Isabel Cristina M Emmerick, Peter Stephens, Dennis Ross-Degnan, Anita K Wagner

**Affiliations:** Department of Population Medicine, Harvard Medical School and Harvard Pilgrim Health Care Institute, Boston, USA; IMS Health, London, UK

**Keywords:** Diabetes, Brazil, China, Thailand, Drug policy, Insulin, Oral hypoglycemic agents, Access to medicines

## Abstract

**Objectives:**

New antidiabetic medications such as insulin analogues and thiazolidinediones have been introduced over the last decade. This study compares the uptake of new agents in three emerging pharmaceutical markets: Brazil, China, and Thailand.

**Methods:**

Using longitudinal IMS Health sales data, we calculated the quarterly percentage market share for types of insulins and oral hypoglycemic agents from 2002 through 2012 in each country. New oral hypoglycemic agents included: alpha-glucosidase inhibitors, thiazolidinediones, dipeptidyl peptidase-4 inhibitors, and non-sulfonylurea secretagogues.

**Results:**

While China had the highest use of insulin cartridges and pens (85.6% in 2010), Brazil was the earliest adopter of insulin analogues and had the greatest use of these products overall (44.6% of the insulin market) in 2010, which then decreased by almost half by 2012. Together, sulfonylureas and metformin dominated the markets in Brazil and Thailand (~89% and ~96% respectively) over the 10-year period. Between 2002 and 2012, there was a shift in use from sulfonylureas to metformin; the market share of newer agents remained 10% or less in both countries. In China, however, market share of new oral agents grew rapidly from 13.1% to 44.4%. While metformin use was relatively stable in China (one-third of the market), sulfonylureas declined substantially over the 10-year period (41.5% to 20.8%).

**Conclusion:**

Given large cost differentials between newer and older insulins and among oral hypoglycemic agents, it is important to evaluate uptake of newer products over time. Uptake patterns differed in the study countries, likely due to different medicines policy approaches. Future research should evaluate how trends in use of antidiabetic products align with national clinical practice guidelines and pharmaceutical policies, as well as the impacts of different patterns of use on cost and clinical outcomes.

## Introduction

Increasing health care expenditures are a common concern worldwide. Increases are associated with several factors, including expanding access to insurance coverage, aging populations, and new expensive medicines and technologies. Every country has a structure of health and pharmaceutical policies that influences patient access to health services and medicines. Middle-income countries with large populations, rapidly growing economies, and large contributions to global pharmaceutical market growth -- often referred to as “pharmerging” markets [1] -- face multiple challenges including growing chronic disease burden, rising consumer expectations for health care, and increasing financial stresses in their health and pharmaceutical sectors.

Diabetes is one of the most common chronic conditions in nearly all countries, affecting approximately 382 million individuals worldwide with another 316 million individuals with impaired glucose tolerance who are at high risk of diabetes [2]. About 80% of people with diabetes live in low- and middle-income countries. All types of diabetes are increasing, particularly type 2 diabetes; the number of people affected by diabetes is estimated to increase by 55% by 2035. Diabetes is a major cause of morbidity and mortality in many countries. Globally, it caused 5.1 million deaths in 2013 and accounted for about 11% of total healthcare expenditures for adults (US$548 billion) [2].

Oral hypoglycemics and insulins are the mainstay of pharmacotherapies for treating both type 2 and type 1 diabetes [2]. While these may be regarded as relatively inexpensive pharmaceutical products by well-funded health systems in industrialized countries, it is not so in resource-limited countries. New oral hypoglycemic medications (thiazolidinediones and dipeptidyl peptidase inhibitors) and new insulin molecules and delivery formulations (insulin analogues, human insulin and insulin analogues administered via pens) have become available over the last decade; these are more expensive than older products (e.g., metformin, human insulins in vials and syringes).

This study compares the uptake of new insulin products and new oral hypoglycemic medications between three middle-income pharmerging countries: Brazil, China, and Thailand. The prevalence of diabetes in Brazil, China and Thailand in 2011 was 9.0%, 9.6% and 6.4%, respectively [2]. During the study period, all three countries have rapidly expanded health and pharmaceutical coverage. Brazil and Thailand have emphasized access to essential generic medicines in their universal coverage programs [3-5]; in China, hospitals’ historical reliance on medicines sales for revenue has created incentives for use of higher cost products [6,7]. Given their different policy contexts, these countries are likely to have responded differently to expanded availability and marketing of newer, more expensive insulins and oral hypoglycemics for diabetes.

## Methods

### Data sources

We used quarterly pharmaceutical purchasing data collected by IMS Health (2002 through 2012). The sales data are generated from reports to IMS Health by multinational pharmaceutical companies and surveys of purchases by hospital and retail pharmacies.

In Thailand, the IMS Health data come from a sample of approximately 200 of about 1100 general and specialized public and private hospitals in the country and 350 retail pharmacies. In Brazil, the IMS Health data represent sales by more than 58,000 pharmacies, based on data from 130 pharmacies (direct sales) and more than 400 wholesalers (indirect sales). Dispensings under the Popular Pharmacy Program [8], a largely private pharmacy network implemented in 2006 to increase access to essential medicines, are captured by the IMS data. In China, the IMS Health data come from a systematic sample of about 15% of hospitals with at least 100 beds (810 general and 208 specialized hospitals) across the country. Approximately 80% of medicines in China are sold in hospitals [9]. IMS Health updated sampling strategies in December 2009 and April 2010 in Brazil and China, respectively, to expand market coverage.

The IMS database classifies medicines according to the European Pharmaceutical Market Research Association (EphMRA) Anatomical Therapeutic Chemical (ATC) classification system, and contains details about the generic drug name, product name, pack size, manufacturer, licensing status, launch date, and volume purchased (sold) in standard units. IMS Health defines a standard unit as the smallest common dose of a product form: one tablet or capsule for oral formulations, one teaspoon (5 ml) for syrups, and one ampoule, vial, or cartridge for parenteral formulations. For insulins, volume purchased was measured in international units.

### Outcome measures and data analysis

We analyzed the use of all antidiabetic medications marketed in the three countries. For each country, we examined use and types and formulations of insulin and oral hypoglycemic agents, from 2002 through 2012.

Insulins included animal and human insulin products, and insulin analogues (genetically engineered insulin products). We also classified insulin products according delivery devices: (a) prefilled pens/cartridges, and (b) conventional vials/syringes. Newer delivery devices (pens and cartridges), although more expensive, may be favored as they can provide more accurate dosing, less pain due to smaller needle gauge, increased social acceptability, better quality of life, and potentially greater adherence [10]. We excluded Exubera, an inhaled insulin, from the analyses; it was used only in Brazil between 2007 and 2008, accounting for less than 0.5% of market share.

Oral hypoglycemics included biguanides, sulfonylureas, alpha-glucosidase inhibitors, dipeptidyl peptidase-4 inhibitors (DPP4 inhibitors), non-sulfonylurea secretagogues, thiazolidinediones, and fixed dose combinations of those molecules. We defined the latter four types as “newer” oral hypoglycemic products since they were more recently brought to market than biguanides and sulfonylureas, which remain the mainstay of oral therapy for diabetes in most clinical guidelines.

We used market share for each product based on market volume as our main outcome measure; we used a similar measure in our previous study [11]. For our analysis, a product was defined as the unique combination of ingredient, dosage form, and strength. Market volume was defined as the number of standard units for oral dosage forms (and international units for insulin) purchased (sold) per 1000 population per quarter. Yearly estimates of the population for each country were obtained from the World Bank to control for population changes over time [12]. Because insulin products could be used by patients of any age, we used total population estimates for analyses of the insulin market, and adult (15 years and older) population estimates for the oral hypoglycemic market. To understand how much a product was used in relation to therapeutic alternatives on the market, we also calculated percentage market share. For example, market share of insulin analogues was defined as the analogue insulin percentage of the total market volume (in international units) for all insulin products in the country.

The study was determined to be exempt from human subjects review by the institutional review board of the Harvard Pilgrim Health Care Institute (Boston, Massachusetts).

## Results

### Insulin products

**Figure 1:**
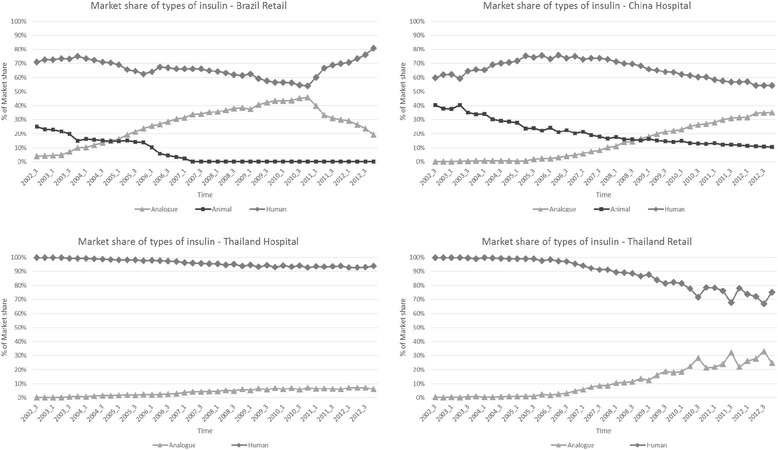
**Market share of types of insulin by country (2002 – 2012).**

Overall use of insulin analogues (regardless of delivery devices), as measured by market share, increased over time in Brazil, China, and Thai retail settings (Table 1) (Figure 1); the increase was very small in Thai hospitals. The increase was steepest in Brazil, although this upward trend reversed sharply starting in early 2011.

**Table 1:** Market shares* of insulin pens/cartridges and vials/syringes by country (2002 to 2012)

	**2002**	**2003**	**2004**	**2005**	**2006**	**2007**	**2008**	**2009**	**2010**	**2011**	**2012**
BRAZIL ***											
Pens/cartridges**	26.17	29.46	34.71	32.75	36.05	38.15	40.37	43.59	46.38	36.71	29.91
Analogue	1.82	3.89	8.56	14.43	21.38	26.53	30.11	33.43	36.47	27.73	19.81
Human	24.34	25.57	26.15	18.31	14.67	11.62	10.26	10.16	9.91	8.98	10.10
Vials/syringes**	73.83	70.54	65.29	67.25	63.95	61.85	59.63	56.41	53.62	63.29	70.09
Analogue	2.11	2.78	4.14	5.68	6.55	7.17	7.20	7.60	8.12	5.32	4.22
Animal	24.16	19.73	15.40	14.39	5.88	0.55	0.00	0.00	0.00	0.00	0.00
Human	47.56	48.03	45.75	47.19	51.52	54.12	52.43	48.81	45.50	57.97	65.87
**CHINA**											
Pens/cartridges**	43.61	48.41	55.81	62.33	67.69	72.71	77.21	80.14	82.86	84.69	85.66
Analogue	0.00	0.33	0.58	1.31	3.58	8.02	14.20	20.35	25.52	30.23	33.92
Animal	0.00	0.00	0.00	0.00	0.31	0.53	0.67	0.62	0.77	0.92	0.52
Human	43.61	48.07	55.23	61.03	63.80	64.16	62.34	59.17	56.57	53.54	51.21
Vials/syringes **	56.39	51.59	44.19	37.67	32.31	27.29	22.79	19.86	17.14	15.31	14.34
Analogue	0.00	0.00	0.00	0.00	0.00	0.02	0.00	0.00	0.00	0.00	0.03
Animal	39.18	36.64	30.33	24.25	21.61	18.08	15.35	14.34	12.72	11.47	10.46
Human	17.21	14.96	13.86	13.42	10.70	9.19	7.44	5.51	4.42	3.84	3.85
**THAILAND HOSPITAL**											
Pens/cartridges**	9.25	11.36	15.92	23.46	31.08	37.30	40.83	47.48	48.26	48.95	51.02
Analogue	0.15	0.23	0.41	1.15	1.98	3.66	4.78	5.84	6.24	6.12	6.66
Human	9.10	11.13	15.52	22.31	29.10	33.63	36.05	41.65	42.02	42.83	44.36
Vials/syringes**	90.75	88.64	84.08	76.54	68.92	62.70	59.17	52.52	51.74	51.05	48.98
Analogue	0.00	0.24	0.81	0.79	0.51	0.44	0.36	0.25	0.23	0.17	0.12
Human	90.75	88.40	83.27	75.75	68.42	62.27	58.81	52.27	51.51	50.88	48.86
**THAILAND RETAIL**											
Pens/cartridges **	15.91	17.12	21.40	25.78	30.18	38.99	44.37	45.44	54.21	59.35	62.02
Analogue	0.23	0.50	0.62	1.32	2.75	6.86	10.36	15.01	21.33	24.21	27.43
Human	15.68	16.61	20.78	24.46	27.43	32.13	34.01	30.43	32.88	35.14	34.59
Vials/syringes**	84.09	82.88	78.60	74.22	69.82	61.01	55.63	54.56	45.79	40.65	37.98
Analogue	0.00	0.00	0.00	0.00	0.10	0.86	1.23	1.26	1.22	0.72	0.72
Human	84.09	82.88	78.60	74.22	69.72	60.15	54.40	53.29	44.57	39.93	37.26

*Market share is the percentage of the total market volume based on volume sold in international units per 1000 population.

**Pens/cartridges includes: CARTRIDGES, P-F PENS, CARTRIDGES RET and P-F PENS RET. Vials/syringes includes: VIAL, VIAL SC and VIAL SC RET.

***Exubera was excluded from the analysis because it is an inhaled insulin thus it does not fit the classification applied; it was used only in Brazil, in the years 2007 and 2008, accounting for only 0.15 and 0.03% of the market share.

Brazil was the earliest and most rapid adopter of insulin analogues. By 2010, Brazil also had a much higher use of insulin analogues overall, accounting for 44.6% of the insulin market, which was substantially greater than use in China (25.5%) and Thailand (6.5% in hospitals; 22.5% in retail pharmacies) at that time. In 2011 and 2012, however, analogue use in Brazil decreased sharply. China was the only country that maintained use of animal insulin products throughout the entire study period, although the rate declined steadily (39.2% in 2002 to 11.0% in 2012). We did not observe any use of animal insulin products in Thailand during the 10-year period, and its use ceased after 2007 in Brazil. Human insulin products dominated the Thailand hospital and retail markets during the study period (Figure 1).

Similarly, pen/cartridge use generally increased over time in all settings. In Brazil, pen/cartridge use increased to 46.4% market share in 2010 (about three-quarters of these were insulin analogues), but decreased markedly from 2011 onwards. China had the highest use of insulin pens and cartridges (Table 1), accounting for 43.6% of the insulin market in 2002 and 85.7% in 2012; about two-thirds of these were human insulin pens/cartridges. In contrast, Thailand had the lowest use of insulin pens and cartridges in 2002 (9.3% in hospitals; 15.9% in retail settings); although lower than Brazil’s in 2002, Thailand’s use of insulin pens/cartridges (in both hospital and retail settings) overtook Brazil’s in 2008, reaching 51.0% and 62.0% in 2012 in Thai hospital and retail settings, respectively.

### Oral hypoglycemic products

In all settings, biguanide use increased and sulfonylurea use decreased over the study period. However, in China there was a clear shift in use from biguanides and sulfonylureas to newer oral antidiabetic medicines.

**Figure 2:**
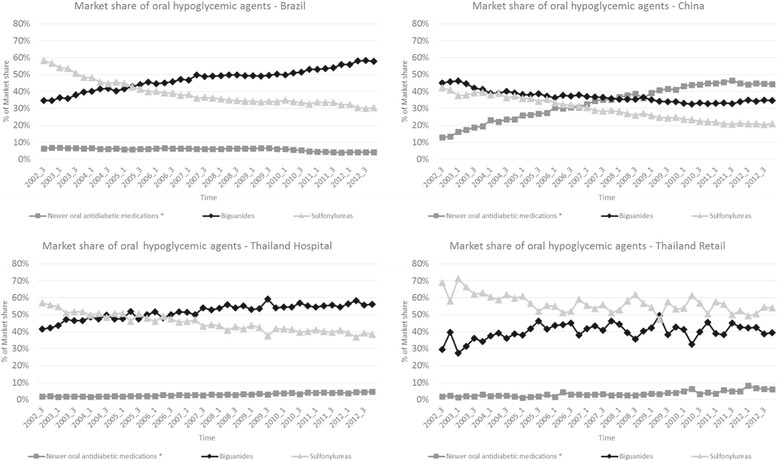
**Market shares of oral hypoglycemic agents by country (2002 to 2012).**

China had the highest use of newer oral hypoglycemic products (Figure 2); market share of these agents grew rapidly from 13.1% in 2002 to 44.4% in 2012. Use of alpha-glucosidase inhibitors accounted for the majority of this higher use (Table 2), followed by non-sulfonylurea secretagogues. While metformin use was relatively stable in China (about one-third of the market), sulfonylureas declined substantially over the 10-year period (41.5% to 20.8%).

**Table 2:** Market shares* of types of oral hypoglycemic agents by country (2002 to 2012)

	**2002**	**2003**	**2004**	**2005**	**2006**	**2007**	**2008**	**2009**	**2010**	**2011**	**2012**
**Brazil retail**											
Alpha-glucosidase inhibitors	2.18	2.12	2.06	1.90	2.03	1.89	1.55	1.45	1.21	0.81	0.62
Biguanides	34.56	37.50	40.94	43.61	45.70	48.61	49.55	49.56	51.39	54.21	57.53
DPP4 inhibitors	0	0	0	0	0	0.77	2.25	2.70	2.48	2.43	2.77
Fixed dose combinations**	1.51	4.35	6.87	8.29	9.04	8.50	9.26	10.07	9.50	8.32	7.60
Non-sulfonylurea secretagogues	3.55	3.58	3.28	2.94	2.76	2.19	1.55	1.25	0.98	0.58	0.33
Thiazolidinediones	0.73	0.86	0.93	1.14	1.55	1.34	0.99	0.93	0.76	0.47	0.46
Sulfonylureas	57.48	51.60	45.93	42.12	38.92	36.70	34.85	34.04	33.68	33.19	30.69
**China hospital**											
Alpha-glucosidase inhibitors	10.50	13.48	16.65	18.41	19.76	21.06	21.95	23.16	25.48	26.94	27.35
Biguanides	45.41	43.48	39.27	38.03	37.34	36.57	35.68	34.31	32.93	33.22	34.64
DPP4 inhibitors	0	0	0	0	0	0	0	0	0.01	0.06	0.15
Fixed dose combinations**	0	0	0	0	0	0.01	0.03	0.15	0.21	0.13	0.15
Non-sulfonylurea secretagogues	2.26	3.85	5.58	6.98	8.31	10.33	12.05	13.72	14.72	15.16	14.13
Thiazolidinediones	0.37	0.66	0.93	1.26	2.35	2.99	3.31	3.79	3.82	3.30	2.79
Sulfonylureas	41.45	38.54	37.56	35.32	32.24	29.04	26.98	24.88	22.83	21.19	20.80
**Thailand hospital**											
Alpha-glucosidase inhibitors	1.37	1.12	1.01	1.02	1.17	1.27	1.29	1.34	1.29	1.16	1.09
Biguanides	41.79	45.92	48.30	49.27	50.45	52.09	54.78	55.11	55.26	55.00	56.63
DPP4inhibitors	0	0	0	0	0	0	0.08	0.23	0.41	0.62	0.71
Fixed dose combinations**	0	0	0.01	0.05	0.08	0.12	0.20	0.31	0.40	0.49	0.68
Non-sulfonylurea secretagogues	0.15	0.15	0.14	0.14	0.16	0.17	0.16	0.16	0.15	0.14	0.15
Thiazolidinediones	0.43	0.54	0.71	0.81	1.18	1.28	1.38	1.61	1.90	2.15	2.33
Sulfonylureas	56.26	52.27	49.83	48.71	46.96	45.06	42.11	41.25	40.58	40.45	38.41
**Thailand retail**											
Alpha-glucosidase inhibitors	1.83	1.58	1.30	1.11	1.98	1.92	1.69	1.73	2.28	1.86	2.75
Biguanides	35.12	32.10	37.81	42.00	42.82	43.16	39.94	43.41	39.71	41.17	40.81
DPP4 inhibitors	0	0	0	0	0	0	0	0	0.16	0.62	1.31
Fixed dose combinations**	0	0	0	0	0	0	0	0.09	0.06	0.05	0.27
Non-sulfonylurea secretagogues	0	0	0.19	0.22	0.38	0.41	0.47	0.50	0.50	0.43	0.50
Thiazolidinediones	0.23	0.50	0.54	0.62	0.66	0.58	0.54	1.43	1.75	1.74	2.37
Sulfonylureas	62.82	65.82	60.16	56.06	54.16	53.93	57.35	52.83	55.54	54.12	52.00

*Market share is the percentage of the total market volume based on volume sold in standard units per 1000 population. IMS Health defines a standard unit as the smallest common dose of a product form: one tablet or capsule for oral formulations.

**Fixed dose combinations include the following: cinnamomum loureirii & glipizide & metformin; glibenclamide & metformin; gliclazide & metformin; glimepiride & metformin; glimepiride & rosiglitazone; glipizide & metformin; metformin & nateglinide; metformin & pioglitazone; metformin & rosiglitazone; metformin & saxagliptin; metformin & sitagliptin; metformin & vildagliptin.

Use of newer oral hypoglycemic products in Brazil was stable from 2002 to 2010, accounting for approximately 6% of market share, with a small (~4%) reduction in 2011/2012. Use of newer oral hypoglycemic products remained below 7% in Thailand throughout the study period (2.0% in 2002 to 4.3% in 2012 in hospitals; 2.1% in 2002 to 6.9% in 2012 in retail settings). Sulfonylureas and metformin dominated the oral hypoglycemic market in Brazil and Thailand over the 10-year period, although there was a relative shift in use from sulfonylureas to metformin in both countries. Between 2002 and 2012, metformin market share increased from 34.6% to 57.5% in Brazil, 41.8% to 56.6% in Thai hospitals, and 35.1% to 40.8% in Thai retail settings.

## Discussion

This longitudinal study showed important differences in uptake trends of newer pharmaceutical products for treating diabetes, a prevalent chronic condition, in three pharmerging middle-income countries: Brazil, China, and Thailand. Our findings suggest that health and pharmaceutical policies of individual countries can have substantial impact on the market share of medications by volume of use and how quickly new agents are adopted in healthcare systems.

China was the fastest adopter of both newer insulin and oral hypoglycemic products. Since the early 1980s and throughout the study period, Chinese hospitals have derived a large proportion (more than 50%) of their operating budgets from drug sales [13,14]. As such, there were strong financial incentives to prescribe newer, more expensive pharmaceutical products [9,13-15]. In 2009, the Chinese government implemented a major national healthcare reform in response to challenges such as increased demands for health care, inadequate health insurance, inefficient use of healthcare resources, and poor quality of care [16,17]. Under the reform, policies were developed and implemented to improve health insurance coverage, care delivery, pharmaceutical supply and access (including through a national essential medicines list for primary care settings which forms the basis for insurance coverage of medicines), and hospital management. One major goal of China’s healthcare reform is to reduce reliance on pharmaceutical sales as revenue sources for hospitals [18]. There are as yet no data on whether this goal has been realized; our data suggest that sales of more expensive therapeutic alternatives for diabetes treatment remain high. China’s higher use of newer alpha-glucosidase inhibitors and non-sulfonylurea secretagogues compared with Brazil or Thailand is in line with its clinical guidelines, which recommend these agents for patients not tolerating or responding to metformin [19]. However, it is unclear to what extent these agents are prescribed as first-line versus second-line therapy for diabetes.

In contrast, Thailand had the lowest use of both newer insulin and oral hypoglycemic products. With the implementation of the Universal Coverage Scheme in 2001 that merged the pre-existing Voluntary Health Card (rural population coverage) and Medical Welfare schemes (poor and indigent coverage), Thailand was among the first middle-income countries to establish such schemes for its entire population [20]. Thailand is also one of the first middle-income countries to adopt a formal process of Health Technology Assessment (HTA) for its coverage decisions; an HTA unit was established in 2002 and the Health Intervention and Technology Assessment Program in 2006 by the Ministry of Public Health [21]. Although Thailand has had a national essential medicines list since 1981, cost-effectiveness, in addition to clinical efficacy and safety, was introduced as a criterion for selecting drugs for the 2004 list [21,22]. Our findings are consistent with a recent study that indicates that human insulin products, metformin and sulfonylureas, which are on the national essential medicines list and covered by the Universal Coverage Scheme, dominate the Thailand market [4]. Newer insulin and oral hypoglycaemic products have only been covered by the Civil Servants Medical Benefits Scheme (which insures about 9% of the population) [23] or self-funded by patients.

The uptake of newer insulin and oral hypoglycemic products was higher in Brazil than in Thailand. Importantly, Brazil was the earliest adopter of insulin analogues and has the most use of these products overall. This finding may reflect the fact that IMS Health data in Brazil represent largely pharmaceutical sales from the private sector where newer products may preferentially be dispensed. Among the three countries included in this study, Brazil was the first to make substantial progress toward increasing financial protection for healthcare expenditures for its population. Since 1989, all citizens in Brazil have been entitled to free health care at primary, secondary, and tertiary levels through a national health system that is funded by taxes and social contributions, such as social security payments [24]. Interest in HTA in Brazil began in the mid-1980s. In 2003, several policies were developed by the federal government to encourage the use of HTA for making clinical, management, and policy decisions [25]. In 2004, Brazil introduced the Popular Pharmacy Program [8] to improve access to essential medicines in the public sector. In 2006, the program was expanded to the private sector and in 2011, selected medicines for diabetes and hypertension became available free of charge at retail pharmacies participating in the program. The Popular Pharmacy Program fully subsidizes human insulin but not insulin analogues; its 2011 expansion may account for the observed shift in use from insulin analogues to human insulin in 2011 [8].

Use of new delivery devices (pens/cartridges) increased over time in all countries, dominating the market by the end of the study period in both China and Thailand. While these devices offer convenience and are preferred by patients [10], over half of these were insulin analogues. We found that insulin analogues accounted for over a quarter of the market share in Brazil, China, and Thailand. These trends still trail high-income countries, where insulin analogues accounted for about two-thirds of the market share in 2009 [26]. In the context of limited resources, choice of insulin analogue pens/cartridges over human insulin pens/cartridges is questionable given the marginal benefits of insulin analogues for type 2 diabetes and their higher prices (3–13 folds higher) [26]. Overall, similar to other published research [26], we found that human insulin dominated the markets in the three countries, although the proportion of analogue insulin use is approaching that of human insulin use in China.

Overall, metformin and sulfonylureas dominated the oral hypoglycemic market in all three countries, which is consistent with global clinical guidelines for diabetes treatment [2] and with national guidelines in the three study countries [19,27,28].

There are notable potential limitations of this study. Our interpretation of the data in China assumes that newer, more expensive products represent higher profits, which may not be correct for all hospitals. The IMS Health data for Brazil only included pharmaceutical sales in the private sector; thus, our analysis does not present a full picture of medication use in this country. We did not have drug price data or patient-level clinical data to evaluate the economic and clinical impacts of the changing trends in insulin and oral hypoglycemic use. Because our analysis was based on pharmaceutical sales, we could not assess the appropriateness of medication use. Lastly, while sampling changes in Brazil and China by IMS Health affected the overall volumes sold for all ATC classes, we calculated market share for a product in relation to therapeutic alternatives on the market. Unless changes in the IMS Health sampling frame differentially affected inclusion of individual products, our market share analyses are robust to sampling changes.

The observed differences in uptake of new therapeutic agents are likely due to complex interactions among multiple actors and factors in country health systems, including government policies to increase medicines access while containing pharmaceutical expenditures, industry strategies to increase access to and market shares of new products [29], clinician prescribing behavior and patient preferences, and patient and system ability and willingness to pay. It remains to be seen how, among other factors, China’s ongoing national healthcare reform, Brazil’s strategies to increase use of chronic disease medicines, and Thailand’s approach to evaluating novel medicines will influence future patterns of use of diabetes treatments. As pharmaceutical markets and health system structures continue to evolve, it is crucial to adapt pharmaceutical policies to generate most health value for the resources spent [30].

## Conclusions

This study compared the uptake of new insulin products and new oral hypoglycemic medications between three middle-income pharmerging countries between 2002 and 2012. Uptake patterns differed markedly in the three countries. China experienced the highest use of insulin cartridges and pens; Brazil was the earliest adopter of insulin analogues, but use of these products subsequently decreased by almost half; use of newer oral hypoglycemic agents remained low in Brazil and Thailand, but market share of these agents exceeded 44% in China by 2012. Observed differences are likely due to different medicines policy approaches.

Future research should evaluate whether medication prescribing is consistent with national clinical practice guidelines, essential medicines lists, and insurance reimbursement lists; quantify the individual and system-wide economic impacts of changes in utilization; and assess population health outcomes associated with different patterns of use over time.
